# Recruitment of caregivers into health services research: lessons from a user-centred design study

**DOI:** 10.1186/s40900-019-0150-6

**Published:** 2019-05-20

**Authors:** Myles Leslie, Akram Khayatzadeh-Mahani, Gail MacKean

**Affiliations:** 1 Department of Community Health Sciences, Cumming School of Medicine, University of Calgary, Calgary, Alberta Canada; 2 School of Public Policy, University of Calgary, Downtown Campus, 906 8th Avenue S.W., 5th Floor, Calgary, Alberta T2P 1H9 Canada; 3 Health Services Management Research Center, Institute for Futures Studies in Health, Kerman University of Medical Sciences, Kerman, Iran; 4 IMAGINE Citizens Collaborating for Health, Calgary, Alberta Canada

**Keywords:** Caregivers, Recruitment, Elderly, Health services research, Patient engagement, User-centred design (UCD)

## Abstract

**Background:**

With patient and public engagement in many aspects of the healthcare system becoming an imperative, the recruitment of patients and members of the public into service and research roles has emerged as a challenge. The existing literature carries few reports of the methods – successful and unsuccessful – that researchers engaged in user-centred design (UCD) projects are using to recruit participants as equal partners in co-design research. This paper uses the recruitment experiences of a specific UCD project to provide a road map for other investigators, and to make general recommendations for funding agencies interested in supporting co-design research.

**Methods:**

We used a case study methodology and employed Nominal Group Technique (NGT) and Focus Group discussions to collect data. We recruited 25 family caregivers.

**Results:**

Employing various strategies to recruit unpaid family caregivers in a UCD project aimed at co-designing an assistive technology for family caregivers, we found that recruitment through caregiver agencies is the most efficient (least costly) and effective mechanism. The nature of this recruitment work – the time and compromises it requires – has, we believe, implications for funding agencies who need to understand that working with caregivers agencies, requires a considerable amount of time for building relationships, aligning values, and establishing trust.

**Conclusions:**

In addition to providing adaptable strategies, the paper contributes to discussions surrounding how projects seeking effective, meaningful, and ethical patient and public engagement are planned and funded. We call for more evidence to explore effective mechanisms to recruit family caregivers into qualitative research. We also call for reports of successful strategies that other researchers have employed to recruit and retain family caregivers in their research.

## Plain English summary

With increasing attention to patient and public engagement in many aspects of the healthcare system, the recruitment of patients and members of the public into service and research roles has emerged as a challenge. Using the experiences of a research project that sought to engage family caregivers in the co-design of technology to better support their work, this paper describes the specific recruiting strategies we used, and identifies general challenges we faced. We describe the successes and drawbacks of various recruitment strategies. We found that recruitment through community-based caregiver organizations is the most effective and least costly mechanism. However, this recruitment work requires a considerable amount of time for building relationships, aligning values, and establishing trust. We identify how existing research funding models may not allow for the relationship building that is central to engaging members of the public in research. These funding models can also force researchers into solving problems they think exist, rather than allowing them to collectively define problems and co-design solutions in partnership with members of the public.

## Background

Family caregivers^1^ are a heterogeneous population [1] who make up a significant unpaid labour force that provides the vast majority of care to elders and others with chronic health conditions and/or physical and mental disabilities [2]. Family care work, driven by demographic and economic change, is on the rise [3] and research into the conditions of, and supports for, this unpaid work is increasing exponentially. Along with attention to policy initiatives addressing labour laws and providing other statutory accommodations for caregivers [4–6] a major focus of both scholars and private industry has been on developing technologies [7] to make caregiving sustainable for family caregivers [8–10] by reducing their burden [11–13]. User Centered Design (UCD) approaches to engaging family caregivers as end users of these technologies are central to this field of research and the agencies that support it such as AGE-WELL NCE in Canada. UCD is a ‘co-design’ or ‘participatory design’ [14–19] process that focuses on partnering with end-users and designing *with* family caregivers not *for* them [20, 21]. Co-design, a mainstream term in health services research, refers to shared leadership and partnership between designers and end-users [22]. Co-design participants are assumed to hold different values, perspectives, and interests with final products emerging from shared visions, co-learning, and mutual understanding [23]. As such, UCD reflects a significant shift in the traditional designer-client relationship as it seeks to engage end users as equal partners with designers in the definition and resolution of particular challenges [24–27].

Although UCD co-design is considered a promising practice, the ethics and practicalities of integrating family caregivers into technology research remain fraught. Although family caregivers may be highly motivated to participate in research, access to these individuals by researchers, especially junior researchers with limited resources, is a known challenge [28, 29]. These family caregivers are generally time-poor [28, 30], with many of them juggling work, family, and caregiving responsibilities [31–33]. Recruiting those who care for persons with Alzheimer’s Disease and dementia can be even more challenging as they are often: socially isolated by their caregiving role and thus difficult to contact; unable to find someone to look after their frail care recipient; and lack access to transportation [31, 34]. Family caregivers’ mistrust of the scientific community and institutions is also reported as a significant barrier to successful recruitment into research [35]. The literature suggests that recruiting these unpaid private citizens into research aimed at improving their lives is difficult indeed [36–40].

Previous research examining the recruitment of caregivers in health services research [36, 41, 42], has tended to focus on minority research participants in the clinical trial setting [36, 43] rather than UCD projects. As the time and work required to identify, recruit and retain these clinical trial participants has increased, the field has turned to professional recruitment coordinators both to liberate lead investigators’ time, and to counter potential participants’ hesitance [44]. Other more recent efforts at professionalization in community and patient engagement for research have focused on creating in-house teams to identify community stakeholders, prepare researchers, and facilitate interactions between researchers and the community [40, 43, 45–48]. Despite these efforts, challenges in recruitment and retention persist [35, 40, 46, 47]. Where advice for UCD researchers has been published, it is highly contextual [40, 48–54] suggesting there are no universal fixes for a universal challenge. There is limited research on the challenges of recruiting caregivers outside the clinical trial environment, leaving those interested in patient engagement and participation in qualitative research with few options.

Similarly, the literature presenting best practices and strategies for successfully recruiting caregivers in UCD co-design research is scant [55]. This is despite the strong emphasis on ‘democratization of research’, and empowerment of patients and members of the public/community as research partners [56]. While this deficit is particularly acute for graduate students with limited resources as well as researchers without grants or university support [57], even early career, or well-established researchers with funding can find recruiting for UCD within the confines of grant development and execution cycles challenging. This paper draws on a specific case study of a particular co-design research project, identifying a number of challenges and counter strategies that we deployed in the course of that project to recruit family caregivers. As a resource for other researchers, we offer an account of the recruiting techniques we deployed, highlighting the time commitment and trust building issues embedded in them. We show how to develop the relationships that are a necessary part of creating the conditions that enable true co-design (i.e. partnering with end-users). We also make recommendations targeting funding agencies interested in the specifics of recruiting for co-design research.

## Methods

The case study at the centre of this paper is a project funded by AGE-WELL Network of Centres of Excellence (NCE). AGE-WELL is a Canadian, federally funded collaborative entity supporting research at the intersection of technology and aging. Our specific UCD project aimed to co-design a smartphone-based application to assist family caregivers providing care for elders. We engaged family caregivers in two in-person Focus Group (FG) meetings, each lasting for two hours, with a gap of at least one week between the first and the second FG. Over the course of the two FGs, family caregivers were invited first to share and discuss their goals and aspirations, and then to imagine feasible technology-enabled supports or paths towards achieving them. As part of our training and mentorship program and to ensure a shared understanding of the research project and to develop trust and relationships, we engaged with family caregivers expressing an interest in our research, through a number of email exchanges prior to convening the FGs. In those emails, family caregivers were given information about the research, their potential role in it, and our expectations. They were also encouraged to ask any questions. At the start of each FG, the lead researcher shared the same information with family caregivers while elaborating on the components of the consent form in detail. We conducted FGs until a total of 25 family caregivers were recruited. Our main recruitment criteria were: 1) being below the age of 65 (with the assumption this population group are more likely to adopt new technology), 2) being a caregiver to a senior citizen over the age of 65 (caregiving for the adults was the focus of the project), 3) living preferably in the Calgary or Edmonton area (driven by the time and costs associated with traveling across the province or country), 4) preferably working, either part-time or full-time (again, assuming this group is more likely to adopt new technology).

In our application to AGE-WELL, we initially proposed to employ a three-step UCD process that included: 1) Citizen panels [35, 46] to discuss and identify caregivers’ needs; 2) Delphi method [5, 31, 58–60] to prioritize identified needs; and 3) Design Thinking method [46] to co-create a basic mock-up of a software solution to the prioritized needs. Both the citizen panel and the design thinking session were to have occurred at a hotel with 25 family caregivers spending a night and day together to define their needs (step one) and co-create a software solution (step three). Unfortunately, it proved impossible to recruit caregivers for this three-step process. While large-group residential sessions have been successful for improving quality in acute care we learned from consulting with our recruitment partners that these arrangements were impractical for family caregivers, many of whom were uninterested or unable to spend such long periods of time away from their care recipient and in the company of strangers. In partnership with Caregivers Alberta (http://www.caregiversalberta.ca/), we altered our methods. This is to say, as part of a co-design process with our community partner, we made changes to accommodate limited schedules and family caregiver preferences for interaction with social acquaintances in smaller groups. Specifically, we abandoned the large group, overnight residential sessions and instead pivoted to meeting caregivers in small groups at places they found more convenient. Methodologically, we set aside Citizen Panels, Delphi, and Design Thinking, replacing them with Nominal Group Technique [61, 62] and Focus Group (FG) discussions [63, 64]. We employed NGT in the first FG to prioritise family caregivers’ goals and used open group discussions in the second FG to explore technology-enabled solutions to those prioritised goals. NGT is particularly well adapted because of its specific focus on empowering all members [65, 66]. It is a stepwise consensus-building democratic process that results in a list of collective priorities [67]. NGT’s facilitated discussions and voting allowed varied family caregivers to contribute equally to group discussions [68].

We engaged a lay representative, who is a family caregiver, as a research partner from the time of grant writing to publication of research findings. In fact, this lay representative is one of co-authors (GM). GM is affiliated by personal and professional experience with family caregiving and the formal healthcare system, and has advised on the content and direction of the research we present in the paper since its inception. GM is involved in an Alberta based citizen-led organization, IMAGINE Citizens Collaborating for Health, who wrote a letter in support of our original grant application to AGE-WELL.

## Results

When we started the project, as with many junior researchers, our knowledge of recruitment strategies was limited [69]. In the grant writing stage we had not adequately anticipated the time, human resources, and costs associated with recruitment efforts [70, 71]. Over the course of our research, from May 2017 through to August 2018, we deployed a number of techniques that we are sharing in this paper with the hope of helping other researchers. Those techniques, employed for recruiting family caregivers who provide care to older adults (see Table 1), included: partnering with intermediary caregiver organizations, using social and print media advertisement, distributing study flyers in geriatric clinics, and employing snowball sampling. We also employed two recruitment support techniques to encourage more participation including remuneration and flexible scheduling.

**Table 1:** Number of family caregivers recruited through different recruitment strategies

Recruitment strategy	No of participants recruited
Caregiver organizations	13
Social and print media advertisement	9
Geriatric clinics	2
Snowball sampling	1
Total	25

### Recruitment techniques

#### Recruiting through caregiver organizations

Our recruitment efforts began with approaches to community-based caregiver organizations- who have the outreach to, and are trusted by, family caregivers- such as Caregivers Alberta, the Alzheimer Society, and other local non-profit support agencies with a focus on older adults. Our central aim in approaching these organizations was to connect and form partnerships with those who had access to the vast and heterogeneous population of family caregivers [71] we sought for our study. In this sense we were engaging the organizations as co-researchers, [72] partnering with them to determine and design how to engage caregivers in the research. As noted above, our partnership with one of these organizations extended to a (co)re-design of the methods originally described in our grant application. To their great credit, grant officers, administrators, and leadership at our funding agency were amenable to, and supportive of these adaptations reflecting the input and priorities of our partner organizations. Initially, we found engaging these organizations for recruitment slow and lengthy. We believe this was due to a combination of missing resources, mutual mis-understanding, and absent trust. We elaborate on this below.

The mandate of these organizations is to support family caregivers. As such, asking them to devote limited resources to rewriting methods, making calls, or sending recruitment emails on behalf of an outside research project was, at an important level, a resourcing issue. Our budget did not include extra resources for these organizations, and as such, we were unable to assist them. Not only were we asking for time and effort on our project’s behalf, we were asking them to ‘sell’ it to family caregivers. This meant organization staff needed not just to understand our project, but to see it as aligned with their own mission. As reported elsewhere, then, we were working to overcome known barriers to recruiting through intermediary organizations which included competing demands on their time, a lack of compensation for their study-related time and effort, and having little vested interest in the study [35]. Trust that our motivations and the potential outcomes of the research were aligned with their own goals needed to be built, particularly as many staff at the organizations saw their first priority as protecting family caregivers by controlling access to them [54, 73]. Once a sense of alignment and trust had been established, and we had maintained a collaborative relationship with these organizations over time [7], our recruitment proceeded successfully. Recruiting through these organizations netted us the majority of participants in our study, 13 of 25, and was the least financially costly [74, 75]. However, the time required to overcome mutual misunderstandings, align goals, and establish trust was significant.

Other challenges associated with this recruitment technique included: a dependence on these organizations for access to potential participants; and an inability to communicate directly with caregivers [42, 76, 77]. While direct communications would have been preferable, the massive heterogeneity and anonymity of the caregiving population made these reasonable compromises in our recruitment process. Similarly, there were compromises inherent in our dependence on caregiver organizations not just for communications, but access at all. As the caregiver population is so heterogeneous, and many of those who provide unpaid care do not, despite meeting formal criteria, see themselves as “caregivers” [35, 46], caregiver partnering organizations are only able to provide access to a limited subset of a much larger population. This is to say, only those who have self-identified as caregivers will ever seek out and become affiliated with a caregiver organization.

#### Recruiting through social and print media

We also extensively exploited social media to recruit caregivers into the study. We designed a Facebook page and posted an ad and targeted several Facebook pages related to caregivers and seniors. We also posted regular notices using Twitter. We used print media too, for instance, our study information appeared on the first page of Dementia Connections magazine. These strategies were the most costly recruitment strategies and were not very effective compared to working with caregiver agencies, as these yielded only nine out of 25 caregivers. Other researchers have reported poor recruitment through these strategies [42], and indeed, few researchers found these means effective [54]. A shortfall of these strategies was receiving many email inquiries from individuals who did not meet study inclusion criteria, a problem experienced by other researchers too [46].

#### Recruiting through geriatric clinics

Our study flyers were also distributed across two geriatric clinics affiliated with the University of Calgary. The flyer included a very brief introduction to the project (in lay language), recruitment criteria, and contact information of the research coordinator. Although the flyer was designed professionally with precise and concise messages, this strategy was not effective as it yielded only two research participants. Our efforts to collaborate with these clinics were not successful which could be due to staff time constraints, bureaucratic rules and procedures, and potential of perceived competing interests as reported elsewhere [78].

#### Recruiting through snowball sampling

We also recruited one caregiver through snowball sampling. This technique has been reported as useful for recruiting research participants who share similar characteristics, and for locating caregivers and elders who may not have access to social services [40, 73, 79]. We used a traditional snowball sampling method by asking successfully recruited caregivers if they know of others who might be interested in participating in our study. We did not take advantage of updated snowball sampling techniques that rely on social media (e.g. retweets, or shared links of study material) rather than word-of-mouth referrals [36, 48].

### Recruitment support techniques

We employed two recruitment support techniques aimed at making participation in the study more attractive to unpaid caregivers with minimal free time. Both support techniques were supplements to our caregiver organization, social and print media, geriatric clinic, and snowball recruitment efforts.

#### Recruitment support through remuneration

We offered two gift cards with the total value of $50 to each caregiver as a token of appreciation for their participation in our study (one card valued at $25 for each focus group meeting). While the ethical case for remunerating patient-participants is clear [36], and other researchers have reported remuneration (e.g. financial incentives/reimbursement, acknowledgment and recognition) to be effective in recruitment efforts [36], it was not obvious that many of our participants were swayed by, or even aware that, a gift card would be provided. Despite inclusion in all of our communications material, a number of participants expressed surprise when the cards were distributed. This diffidence may have reflected an understanding of our study as an extension of their otherwise unpaid caregiving work, or a desire to avoid seeming preoccupied with money, but in any case the gift cards did not appear to have been major factors in our recruitment success. We were unable to measure their value in building the project’s reputation as ethically sound and genuinely valuing caregivers’ experience and perspectives. Beyond our own research, our assumption is that providing gift cards may contribute to participants developing a positive view of research, making them more likely to get involved in the future, and to speak positively about their experience with others [80]. We argue these longer term ‘legitimacy’ benefits should join ethical arguments in favour of research budget being devoted to providing remuneration for participants’ time.

#### Recruitment support through flexible scheduling and meeting location

To facilitate caregivers’ attendance, especially working caregivers, we held focus group meetings outside normal business hours and in locations suited to most of them [81]. We traveled to Cochrane (a town located 18 km west of the Calgary city) for two focus groups, held another set of two focus groups in Edmonton, with the balance were held at the School of Public Policy, located in downtown Calgary, a location easily accessible by various form of public and private transportation.

## Discussion

Having described our experiences and efforts at recruitment, and recruitment support, in a UCD co-design project targeting improvements in the lives of family caregivers, we move to more general observations and recommendations in this section. There are still many obstacles in the recruitment of family caregivers, patients, and members of the public in research that prevent their meaningful engagement (for a summary of these challenges see Table 2). Our findings suggest that a true co-design - where research participants are seen as equal research partners and the authority to define and act on problems is shared with them – requires structural changes. True co-design requires meaningful engagement with both family caregivers, patients, or citizens, and the organizations that may assist with their recruitment at the moment research is first contemplated. Carman et al. [82, 83] have characterized this engagement as operating across a spectrum of volume that is reminiscent of Arnstein’s [49, 84, 85] earlier view of citizen participation. Their volume continuum ranges from low level *consultation*, through mid-level *involvement*, and up to high level *partnership*, with patients afforded increasing presence and authority at each level. Without a shared sense that the research is a high priority for all stakeholders (family caregivers, trusted organizations, and researchers), participation will only really occur at the levels of consultation or involvement. This is to say, it will not be true co-design, which we understand requires engagement at a partnership level.

**Table 2:** Summary of challenges associated with recruitment in co-design research

Research Phase	Challenges	Actions	Costs
Grant writing	- Limited experience with co-design research - Limited knowledge of recruitment strategies - Limited anticipation of time, human resources, and costs associated with recruitment	Engaging one family caregiver on the research team at the time of grant writing - Self-training the co-design research within limited time	- Stress and time pressure especially close to the grant submission time
Planning for data collection	- Limited familiarity with family caregiver’s context and realities - Non-feasibility of research methods in the original grant proposal	(Re) co-designing methods with trusted community caregivers organizations	- Traveling costs to visit community partners - Time to build trust with community partners
Data collection	- Ensuring shared understanding and building trust - Accommodating family caregivers’ preferences for time and locations - Prolonged data collection process longer than anticipated	- Frequent email communications with interested family caregivers prior to FGs - Seeking extension from funder	- Time - Long distance travels - Working with limited financial and human resources
Recruitment Strategies			
Partnering with caregiver organizations	- Slow and lengthy process due to missing resources, mutual misunderstanding, and absent trust - Misalignment of values, roles and expectations	- Constant communications with to build trust and to (re)co-design the research methodology	- Human, time, and financial resources
Social and print media	- High cost compared to least effectiveness - Receiving many irrelevant email inquiries	- Designing professional social and print media	- High financial cost - Time
Flyers in geriatrics clinics	Clinic staff time constraints, bureaucratic rules and procedures, and potential of perceived competing interests	- Designing professional yet lay-public friendly flyers	- Time and financial resources associated with designing professional flyers
Traditional snowball sampling	-Least effective in recruiting - Relying on word-of-mouth referrals	- Seeking recruited family caregivers to engage other family caregivers	- Time

A number of research funding agencies across the world including CIHR in Canada, and Patient–Centered Outcome Institute (PCORI) and Quality Enhancement Research Initiative (QUERI), both in the United States, and INVOLVE (a project established by the British National Institute of Health Research to increase public engagement in research) in the United Kingdom are now encouraging co-design research through partnership between researchers and other stakeholders including members of the public. The CIHR Strategy for Patient-Oriented Research (SPOR) is another funding partnership that CIHR formed to make co-design patient-oriented research a reality in Canada. In Alberta, the home jurisdiction for the case study at the center of this paper, Alberta Innovates - a provincial research funding agency - has introduced Partnership for Research and Health Innovation in the Health System and the Collaborative Research and Innovation Opportunities program to encourage partnership between researchers and community members. Despite these efforts, we argue that some existing funding models and grant processes tend to force researchers into solving a problem that they think exists, rather than allowing them to co-define problems and co-design solutions alongside participants. This is due, in part, to the emphasis on ‘problem solving’ rather than ‘problem definition’ or ‘problem structuring’ [86, 87] which is one of the key reasons for policy failure [40, 88]. To overcome this, we argue that granting organizations should consider seed, or incubation grants that facilitate not the presentation of pre-made research questions to nominally engaged patients, but rather the trust building and value aligning that are necessary for co-design. An example of such funding initiative is the Coalitions Linking Action and Science for Prevention (CLASP) led by the Canadian Partnership Against Cancer that offer allowances to researchers to build relationships and coalition and partner with stakeholders as a granting prerequisite. Clearly there is space, and funding, for traditional research models as well, but structural supports – in the form of time and money – for the participatory action model we are advocating here are long overdue. If we are to take engagement, at the point where it distributes the most power to patients, caregivers, or citizens seriously, reforms to granting cycles and content ought to be considered carefully by research funding agencies.

If we are to avoid the marginalization of co-design in health services research, and reap the benefits of partnership-level engagement, we suggest the following participant, and organization level changes in the funding structures: 1) At the participant level, provide seed grants for working on research proposals with patient/caregiver partners, and factor in relationship-building periods at the start of grants, 2) At the organizational level, include opportunities to build in funding to support the often small non-profit patient/caregiver organizations that researchers want to engage in their co-design/co-research processes.

## Conclusions

Despite being best practice and an ethical imperative, patient and public engagement in health services research generally, and the recruitment of participants for UCD co-design research remains a persistent challenge. Through employing various strategies to recruit unpaid family caregivers in a UCD project aimed at co-designing an assistive technology for family caregivers, we found that recruitment through trusted caregiver agencies is the most efficient (least costly) and effective mechanism. The nature of this recruitment work – the time and compromises it requires – has, we believe, implications for funding agencies; they need to understand that working with caregivers agencies, requires a considerable amount of time for building relationships, aligning values, and establishing trust. In addition to being time and relationship intensive, the compromises inherent in working with caregiver organizations mean that access to the full family caregiver population, and so a ‘representative’ sample, is not a realistic expectation for funding agencies. These factors are not readily known by young researchers in the grant writing stage, which lead the research project to extend beyond the funding timeline. Moreover, effective engagement of patients/caregivers and their partnering organizations requires political support for the genuine devolution of power and decision making to patient/caregiver organizations and to the citizens and caregivers with whom they engage. Some of what we learned about engaging family caregivers and caregiver partnering organizations in research might be transferable to engaging other groups as well. We call for more evidence to explore effective mechanisms to recruit family caregivers into qualitative research. We also call for documentation and reporting of the successful techniques that other researchers are employing to recruit and retain family caregivers in their co-design, partnership-level research.

## Abbreviation

FG: Focus Group
NGT: Nominal Group Technique
UCD: User-centred Design

## Footnotes

1 Family caregiver is defined by Family Caregiver Alliance as “any relative, partner, friend or neighbour who has a significant personal relationship with, and provides a broad range of assistance for an older person or an adult with a chronic or disabling condition.”
